# Ricord on Venereal Diseases

**Published:** 1846-02-01

**Authors:** 


### Ricord on Venereal Diseases.

*A practical Treatise on Venereal Diseases, &c., by P. H. Ricord, M. D., Surgeon of the Venereal Hospital of Paris, &c., translated from the French by Henry Pilkington Drummond.—Lea & Blanchard, Philadelphia*, 1843.

*A complete practical Treatise on Venereal Diseases, and their immediate and remote consequences, &c., by William Acton, late externe at the Female Venereal Hospital, Paris.—London*, 1841.

We have placed the title pages of two recent works on Venereal Diseases at the head of this article, with the design of condensing therefrom a brief synopsis of the latest, and most approved pathological and therapeutical principles relating to this department of our science.—Foremost among those who have investigated with distinguished success this class of maladies, stands Ricord. Possessing immense opportunities for observation, in the wards of a large Hospital specially devoted to these diseases, and in an extensive private practice which his reputation and position have secured to him, he has not lost sight of scientific objects, but has continued the prosecution of his researches with a degree of zeal and ability worthy a faithful votary of science. In this specialty Ricord occupies a position with which there is scarcely a parallel in any other department of medicine. He may be said to represent, in his opinions, the present condition of the pathology and therapeutics of this branch of nosology. We have thought, that by giving such a synopsis as we have proposed, we should render an acceptable service to our readers, from the fact, that, in general, practitioners do not meet with a sufficient number of venereal cases to prosecute continued investigations for themselves, nor to stimulate them to much attention to the literature of this particular department; and, yet, all are liable to meet with cases of importance, which may involve questions of great nicety, and be attended with not a little responsibility. In making the synopsis, we shall avail ourselves almost exclusively of the two works referred to in the caption. The first is a translated work of Ricord himself, the greater portion of which is devoted to his researches upon inoculation. The second is written by a pupil of Ricord, and the object is, mainly, to make known the results of his investigations to the English reader, which, however, the Author's own observations have enabled him to confirm. It is an English work, which has not, to our knowledge been republished in this country.* It is accompanied by a volume of plates, exhibiting the pathological appearances of the various forms of these diseases, which rival, in beauty of execution, the splendid engravings lately published by Ricord himself.—In giving an analysis of the contents of these works, we are compelled, in order to adapt ourselves to our narrow limits, to omit much that we should be glad to embrace if we were not restricted in space. We shall confine our attention, chiefly, to those points which are of practical interest or importance. Our object, as already stated, is to render a labor-saving service to those of our readers who have not time or opportunity to bestow perusal upon the works themselves; but, in addition to this, we trust that the incompleteness of our analysis may determine some to go

* Since this was written it has been issued from the press of J. S. Redfield, New-York.

directly to these works for information, who otherwise would not. We shall treat of the subject in two successive numbers, devoting attention in the present number to that class of venereal affections, usually entitled *Gonorrhœa.*

Ricord rejects altogether the use of the term *Gonorrhœa.* It has, as all must admit, no etymological significancy, and there is nothing but the sanction of usage to be quoted in its favor. Ricord, however, is led to reject it from certain pathological views, which, we presume, will strike some of our readers with surprise. He does not regard the disease commonly called gonorrhœa to be essentially different from that known as leucorrhœa in the female. In fact, he classes them together under one appellative, viz: *Blennorrhagia*—signifying, etymologically, flow of mucus. We will not discuss the appropriateness of the title. It is certainly preferable to that which signifies a flow of semen, and, perhaps, is the best which could be adopted. Of the pathological principle just stated, we will say a few words. He does not regard Blennorrhagia, in any form, as involving any thing *specific*—a peculiar *virus* in other words. It is defined to be a "simple inflammation of the mucus membrane, a consequence, more or less direct, of sexual intercourse, not necessarily, although often contagious." Of the circumstances which give to the products of this inflammation a contagious property in some cases, while, in others, it is devoid of this property, we do not find much discussion in these works. The doctrine as thus stated, if we do not err, is quite at variance with the opinions which are generally entertained by practitioners in this country. The latter recognize a distinction founded chiefly on the hypothesis that the property of contagion is dependent on *something specific.* Ricord regards the disease, when propagated by contagion, not produced "by any thing specific in the muco-pus, but as a consequence of simple irritation;" in fact, as from "any chemical irritant." We confess we are not prepared to accept this doctrine fully, even on the authority of Ricord. Of course it would be absurdly presumptuous to provoke any comparison of data upon which individual conclusions have been drawn, yet facts which have occurred under our own observation (as they have under the notice of every practitioner) strike us forcibly as opposed to this view. All medical men know how common leucorrhœa is among married women; probably not one in ten escape it altogether, during a period of five years of married life. We have in a few instances known an urethral discharge in the husband fairly attributable to this source, but, if the views of Ricord are correct, ought such cases not to be vastly more frequent than they are? As a matter of statistics, which we can to some extent estimate, compare the number of individuals who are constantly exposed to the contagion of leucorrhœa with impunity, with the number who become affected from impure sexual intercourse. The difference of ratio in the two instances is immense, and we do not see how it is to be accounted for, unless we admit the existence of a distinct poisonous principle in the one case which does not obtain in the other. In this comparison, we are to take into account the use of precautions which are probably as common in the latter case, as they are seldom resorted to in the former; and, also, another fact, which, in so far as our observations have extended, has been invariable, viz: urethral discharge due to leucorrhœa is comparatively a slight affection, yielding, in all cases, readily to simple medical treatment.

We should remark here, that Ricord does not admit any identity of essence between Gonorrhœa and Syphilis. The former he distinguishes as Syphiloid affections—resembling Syphilis. The grand test of the distinction is inoculation. It is here that the researches of this distinguished observer have been eminently useful to science. We shall speak more particularly of this when we come to treat of Syphilis. In this connection it will suffice to say, that notwithstanding numerous experiments, made in every variety of mode, no specific results have been produced by the introduction of gonorrhœal discharge into the cellular tissue. This furnishes a radical distinction between it, and the products of a truly syphilitic affection. It has a peculiar affinity for mucus surfaces. The conjunctiva and rectum may take on consecutive disease from direct contact; the buccal and nasal surfaces, however, are not known to have become thus diseased. We might adduce facts appertaining to *gonorrhœal* ophthalmia in support of the views commonly entertained on the specific quality of gonorrhœa, but this would lead us into too extended a discussion.

If the principle just referred to of Ricord be not correct, there is a manifest impropriety in including all discharges directly or indirectly consequent upon sexual intercourse, under the same generic term. The distinction now intended to be conveyed by the terms *gonorrhœa* and *leucorrhœa*, should be maintained, but other names might doubtless be selected more appropriate. In expressing our opinion of an essential distinction, we are free to admit that the diagnosis between the two is not easy, nor, in all cases practicable; nevertheless, this is not conclusive against such a distinction, provided there be a wide difference in results, and in other circumstances not immediately appreciable by the medical observer.

One of the most important of the demonstrations of Ricord relates to the fact that a gonorrhœal discharge to all appearance, in some instances has been known to produce syphilis, and to develope the usual characters on inoculation. This fact, according to Ricord, is conclusive evidence of the existence of a chancre within the urethra, which occasions a gonorrhœal discharge as a complication. The practitioner, will, at once, perceive the importance of close examination of the urethra in all cases of apparent gonorhœa. He will, also, see the necessity of caution in stating to a patient the nature of the risk incurred in exposing another individual to infection. In cases where it is important to demonstrate the presence or absence of the syphilitic virus, we may resort to inoculation of the same individual as an unfailing test. To gonorrhœa complicating chancre he gives the name of *virulent gonorrhoea.*

Whether we admit that the contagion of gonorrhœa generally involves the presence of a virus of a specific nature,—although different from the syphilitic virus—or not, the importance of being able to distinguish the circumstances characterizing a more or less degree of communicability, is equally apparent. Here seems to be a blank in the history of this class of diseases which remains to be filled by future researches. Farther observations, either with the microscope, by chemical analysis, or by some method analogous to inoculation in syphilis, may discover satisfactory means of practical discrimination. At present, it must be confessed, we have no demonstrable tests.

The interval of time which elapses between exposure and the development of the disease, is a point of considerable practical consequence. Ricord believes that in all cases a few days only elapse. He does not admit a period of *incubation;* cases apparently controverting this position, he explains, either by attributing them to a new infection directly or indirectly received, or to causes which do not involve contagion. The case given by B. Bell in which it became developed sixty days after sexual intercourse, he discredits, or regards it as susceptible of explanation irrespective of the sexual exposure.

In speaking of the origin or reproduction of gonorrhœa (blennorrhagia) from other causes than sexual intercourse, Ricord remarks as follows with reference to Asparagus: "I have seen more than one person attacked with gonorrhœa annually in consequence of eating asparagus, and on leaving off this vegetable the discharge has ceased." This suggests an obvious caution in the dietetic management of cases of this disease.

*When is a patient secure against communicating the disease?* Few practitioners of experience but have had questions addressed to them with earnestness on this subject. There are cases in which it is of great importance to be able to give a definite answer to such questions. We will quote Ricord's opinion on this topic, and the quotation will also embrace his views of the conduct which the medical adviser is bound to pursue in certain cases of peculiar delicacy and responsibility. Mr. Acton says, "Ricord considers that when the secretion is reduced to a thready mucus, which is transparent, like vermicelli, contagion is not to be dreaded. On the contrary, so long as the secretion is purulent, it is capable of causing a similar complaint in any mucus membrane with which it comes in contact." * * * * "Patients sometimes present themselves to me to know if they may marry. I persuade them against it if they have a simple gonorrhœa; but if it be a virulent complaint,—i. e. Syphilitic—I wash my hands completely of the affair. If they still persist, I tell them that they may give a gonorrhœa to their wives, which, unless cured previously to confinement, may cause a loss of eyesight to the child:—called, however, afterward to cure the lady, I attempt to explain the affection which she has contracted, by speaking of the fatigue of the honey moon, as well as the *dejeuner a la fourchette*, and, in the interim, cure both parties, of course forbidding connection. Such is the part that a medical man often has to play, and many disputes in married life may be thus avoided, and the Surgeon must lend himself to deception."

We pass now to the treatment. This, Ricord divides into 1st, the Prophylactic, which we omit; 2d, the *abortive*, in other words, treatment with a view to strangle the disease before its full development; 3d, the curative, properly so called. This branch of the subject is treated quite comprehensively in the work by Ricord. The style is, however, so close that it is difficult to condense. We must omit details which are of minor consequence.

He first considers the treatment of the disease in the female, and here the reader is to bear in mind that he refers to all blennorrhagic discharges, embracing both leucorrhœa, and gonorrhœa proper. The disease may involve in the female the following parts, alone or combined: vulva, vagina, uterus, and urethra. This leads us to speak of the speculum, which is so freely employed in Paris. Excepting in active inflammation of the parts the advantages of an ocular inspection are obvious. It enables in certain cases to determine the exact location and character of the lesions upon which the continuance of the disease depends. Motives of delicacy have prevented its adoption, to much extent, in this country, in private practice. Mr. Acton says that the inmates of the Venereal Hospital for females once rose in mutiny against it, but were finally reconciled; and, now, Ricord states that he frequently receives notes from the *elite* of Paris requesting his attendance, with a postscript intimating that his speculum will be required. With regard to its use among us, we believe there are extremes on either side to be avoided. A modest female should not be desired to submit to that violation of feminine delicacy which the use of this instrument requires, for mere scientific curiosity, nor unless important information is to be expected from this source; and on the other hand, where it is necessary to a satisfactory diagnosis, and effective treatment, it should be insisted upon as an indispensable condition of assuming the responsibility of the case. There can be no doubt that it is not sufficiently used in this country. We cannot, we confess, speak of its value from much personal experience, but those of our friends who have been more faithful in this respect than ourselves, have assured us that they have not encountered that degree of opposition, or repugnance, which we might be led to expect. Of course much will depend upon the manner in which it is proposed, and the subject presented to the patient.

During the acute stage, in the female, Ricord recommends absolute rest, abstemious diet, general baths, with local injections, while in the bath, of the same water. Leeches may be applied on the exterior of the greater labia, or on the fold of the thigh. The local remedies are cleanliness, and slightly narcotic fomentations. The diseased parts may be *isolated* by introducing tampons of fine lint dipped in emollient, narcotic fluids. If the uterus be affected, emollient cataplasms may be applied to the hypogastrium. He never applies leeches to the cervix uteri. Strangury, dependent on *urethritis*, is often relieved by a tampon imbibed with a solution of belladonna.

An affection of the ovarium, analogous to orchitis in the male, is believed by Ricord occasionally to occur as a complication in the female.—This is to be treated by antiphlogistic measures commensurate with the intensity of the symptoms.

The *abortive* treatment, as he styles it, rarely obtains in women, from the fact that they seldom apply very early in the disease. In this respect it is far different with the other sex. During the acute stage, when the disease resists the measures just mentioned, Ricord states that he sometimes obtains astonishing results from the use of nitrate of silver. This he applies by means of the solid stick, or by lint dipped in a solution, introducing, in the former case, dry lint after the cauterization. He never prescribes copaiva, or cubebs, deeming them either hurtful, or without effect. He never administers mercury in the acute stage, and very rarely afterward.

After the acute stage has passed, he considers it important to hasten the cure, so as not to allow the chronic state to become established. For this he depends on injections, chiefly. He prefers over all others, solutions of the crystalized acetate of lead, or of sulphate of alum and potassa. The strength of the solution is to be increased to an ounce to a pound of water.

He adds, that, "by means of injections, and tampons dipped in the same liquids, sixty out of a hundred patients will generally be cured in the space of from twenty days to two months, including the treatment of the acute stage."

When it becomes chronic, however, it may not yield promptly. The question will now arise whether the tissues have undergone change.—This is to be ascertained by ocular inspection. If no lesions have occurred he employs applications of a more tonic character, as decoction of oak bark, with equal quantity of solution of the sulphate of alum, zinc, extract rhatany, sublimate, &c., successively. He attributes much efficacy to *isolation* of the diseased parts, as he terms it, by moderately distending the vagina with dry lint. When the internal surface of the uterus is the seat of the disease, he injects this organ with a solution of the nitrate of silver, six grains to the ounce. As this ought to remain but a short time, he employs a syringe with two pumps and separate canulas, by which he first injects the solution, allows it to remain a minute or two, and then forces in pure water to wash the surfaces.

Ulcerations, or papular granulations must be cauterized with nitrate of silver, or with nitrate of mercury, previously cleaning their surfaces with a piece of lint. Where the ulcerations have destroyed the tissues, however, to much depth, the caustic must be cautiously used. Sprinkling calomel and covering with dry lint, will, then, sometimes succeed.

Copaiva and Cubebs appear to have no decided action upon the disease affecting the vagina or uterus, while upon the urethra, as in men, it has a powerful effect.

After the cure of the disease, he recommends injections of cold water, to be continued for some time, excepting shortly before and after, as, of course, during menstruation. He adds some useful instructions respecting the mode of using local applications, but these our limits will not allow us to notice.

*Treatment of Gonorrhœa in the Male.*—The affection in the male consists of two varieties, dependent on its location, and the two may be presented singly, or combined. When the discharge emanates from between the prepuce and glans, constituting what has been called external gonorrhœa, bastard-clap, &c., Ricord distinguishes the affection by the title of *balanitis*. The treatment which he finds most effective for this variety, is to cauterize slightly the diseased surfaces with the nitrate of silver, afterward placing a bit of dry linen over the glans. Subsequently to the operation, compresses dipped in cold water, or diluted liq. plumbi, should be applied. When this form of the disease is complicated with phymosis, some difficulty may arise in consequence of not easily getting access to the diseased surfaces. Whenever practicable, the cauterization will be found most effectual if made with the solid nitrate; when this cannot readily be done, a solution may be used as an injection. "In the treatment of balanitis, neither mercurials, copaiva, nor other anti-gonorrhœal medicaments are required."

The foregoing is a somewhat rare variety. The disease usually affects the urethra and this alone, constituting *urethral blennorhagia*, according to Ricord's arrangement, or *urethral gonorrhœa*, or *gonorrhœal urethritis*. The treatment relates first to the *abortion*, or *strangling*, to use the hyperbolic language to which the French are prone in the application of epithets. Ricord advocates the principle that the disease is to be cured as speedily as possible—that no danger is to be apprehended from the sudden arrest of the discharge by appropriate measures, but, on the other hand, the unpleasant symptoms, and consequences, are in direct proportion to the duration of the disease. This is an important principle, and one which is not, we imagine, generally recognized by practitioners.—To cut short the disease if practicable, is, frequently, if not generally deemed injudicious, inasmuch as it is considered to involve a greater liability to accidents—such as swelled testicle. According to Ricord's view, however, this, and other accidents are far less liable to occur if the full development of the disease can be prevented, or, if developed, it be speedily cured. We have, then, his authority to adopt measures without delay to arrest the disease, if possible, before it attains its heighth, and under all circumstances to adopt that course which will cure the disease in the shortest period, provided the course, in itself, be unattended with risk of injury.

The measures taken to arrest the disease are to be adapted to the condition of the parts, as respects pain, and other indications of acute inflammation. If acute symptoms have developed themselves before the case comes under treatment, twenty or thirty leeches may be applied to the perinæum, and copaiva, or cubebs administered in pretty large doses, so as to produce a perturbatory effect, or a sudden revulsion. Frequently by this treatment conjoined with rest, low diet, suspension, &c., the discharge will stop in 3 or 4 days, or, if the disease be not cut short, its progressively acute character will cease, and by continuing the treatment it will terminate on the 20th or 21st day. He does not advise injections at the commencement if pain and other acute symptoms are present. On the other hand, if these symptoms are absent—if the disease do not commence with them, or the patient applies before they have time to be developed, injections may be pursued with much expectation of success, and without any fear of ill consequences. The means proposed by Carmichæl of injecting a solution of silver, 10 grains to the ounce, he does not approve of. It will often succeed: but the surgeon plays at a game of double and quits; if it fail, it may do harm in addition to the failure. He recommends, in lieu of this, a very weak solution, ¼ gr. to the ounce of distilled water, and eight single injections to be made, at regular intervals, with a glass syringe, during the first 48 hours. The cubebs, or copaiva, to be given in conjunction, and continued afterward. By pursuing this course, Mr. Acton declares that in 50 out of 100 cases the disease will be immediately checked, without fear of swelled testicle, or stricture.

When in spite of appropriate measures at the commencement, the disease passes into a chronic form, injections of liq. plumb. may be employed for 6 or 8 days, and if no results are produced, the nitrate of silver may be again tried, and, successively, other astringents, such as alum, zinc, &c. When a whitish gleet only remains, injections of red wine holding in solution pure tannin (3 grs. to ℥) will often prove effectual. The anti-gonorrheal medicaments may also be employed. These embrace more especially, copaiva and cubebs. The former he places first in efficacy. He prescribes it pure when it will be borne, in doses of from ʒi to ℥i two or three times daily. The cubebs he gives in doses of ℈i to ʒii, from one to four times daily.

For the details of the management of the varieties and phases of the disease we must refer to the work of Ricord. The instructions are given with remarkable conciseness, so that it is impossible to do justice by an analysis without quoting the greater part of this portion of the treatise. His general views with regard to injections are specially important. Many practitioners of late years have abandoned their use almost entirely, from an impression that they are liable to induce stricture. Ricord attributes this result rarely, if ever, to the effect of injections, but to the duration of the complaint. Hence to prevent organic changes, he endeavors, by this mode, to cure the disease as rapidly as possible. He considers the apprehension of danger from repercussion utterly groundless.

During the continuance of the disease, and for some time afterwards, he recommends abstinence from the warm bath, as he has observed this to increase and even recal the discharge. We have had occasion to observe this fact.

Cases of Gonorrhea, as almost every practitioner is able to testify, will occasionally be found to resist every method of treatment by medicaments, and locally, by various injections. In these cases it is to be presumed there is some permanent difficulty in some portion of the urethra, and this canal is to be carefully explored to determine its location and character. This exploration will probably discover either increased sensibility at some portion, or stricture. If the former, Ricord adopts the practice recommended by Lallemand, of applying the solid nitrate of silver. Of the treatment of stricture, we must refer the reader to this and other authors on the subject. We shall also omit an account of the pathology and treatment of swelled testicle, or, as Ricord terms it, *gonorrhœal epididymitis*, except to notice that when this complication exists, it does not prevent him from treating the urethral discharge by injections, &c.; and, also, a method which appears to be original with himself, viz: compression. He says that by means of compression he obtains the cure of sympathetic epididymitis in five or six days, prevents the development of hydrocele, and permits patients to follow their occupation without any ill effects. This practice is new to us. We give his description of its application in order that our readers may be induced to try it, as, of course, they will be ready to do on the authority of Ricord:

"Compression is applied by means of bandages of emplast. c. hydrarg. about half an inch in width. The diseased testicle being carefully held, so as not to occasion too much pain, is to be turned toward the lower part of the scrotum, without distending the cord, at the same time separating it from that on the other side. The strips should then be applied in circles, beginning by placing the first on the insertion of the cord, and sufficiently firm to prevent the organ from slipping. This being done, the circles should be continued around the testicle, so as to produce a considerable, but uniform pressure, avoiding as much as possible making any folds in the skin. Beyond this point, separate straps should be applied, so as to exercise a pressure from above below, and thus forming a kind of basket." He adds, "if this dressing is to succeed, the sufferings of the patient will diminish from the moment of its being applied, and, at length, entirely cease. If this be not the case, it must immediately be removed."

Other collateral subjects connected with Gonorrhœa, such as gonorrhœal ophthalmia, phymosis, paraphymosis, &c., we must also pass by.—We have endeavored to give a succinct account of the prominent and peculiar views of Ricord, as developed in the two works placed at the head of this article, in so far as they relate to the subject of gonorrhœa in general. In our next number we purpose to resume the analysis, presenting, in the same manner, his opinions on the subject of Syphilis.

